# Adolescent digital intimate partner violence: A call for global policy strategies

**DOI:** 10.1016/j.ghrp.2026.10.007

**Published:** 2026-10-30

**Authors:** Taren L. McGray, Thao Ha

**Affiliations:** Department of Psychology & Reach Institute, Arizona State University, 950 S McAllister Avenue, Tempe, AZ 85281, United States

**Keywords:** Digital Intimate Partner Violence, Adolescent Dating Violence, Technology-Facilitated Violence

## Abstract

Digital technologies have become a fundamental part of adolescent communication and romantic relationships across the globe. While technology enables adolescents to feel more connected, it also introduces new ways to perpetrate and experience intimate partner violence (IPV). This paper synthesizes emerging evidence about global digital IPV to critically examine the current state of knowledge about adolescent digital IPV and make recommendations for the incorporation of digital IPV into global-level frameworks. We identify that although there are many similarities to in-person IPV, unique digital affordances, such as absence of interpersonal cues, lack of immediate social feedback, remote surveillance, permanence, and anonymity, create a new context to perpetrate and experience IPV, which may exacerbate psychological and developmental harm and complicate prevention and intervention. Despite this, digital adolescent IPV is missing from global frameworks that critically influence policy, funding, and research. Global frameworks should incorporate the unique challenges of digital adolescent IPV by establishing a specific taxonomy, creating a global research agenda, and developing guiding principles for prevention and intervention. Recommended guiding principles for policy and practice include prioritizing recognition of adolescents as a high-risk population, respect for adolescent participation in digital platforms while teaching digital competence, co-creation with adolescents, and partnership with technology platforms to affect change at the design-level. This paper concludes that global frameworks for digital adolescent IPV are critical in order to mobilize coordinated research, evidence-based programming, and adolescent-centered policies to combat digital IPV and develop technology to foster resilience and healthy relationship development worldwide.

## Introduction

Digital technology has transformed how adolescents initiate and maintain romantic relationships through social media, video calls, and instant messaging.^1^ Although digital connectivity has improved access to social support, it has also introduced new modalities for intimate partner violence (IPV) during a formative developmental period when adolescents begin to learn and form relationship patterns, which has potentially lasting effects on future relationships and health outcomes.^1^, ^2^ Digital IPV is defined as the use of technology to remotely perpetrate sexual coercion, psychological harm, threats of physical aggression, and controlling behaviors between current or former romantic partners.^2^, ^3^ There is potential for digital IPV wherever in the world there are adolescents who date and have access to technology. Adolescent access to internet ranges from 50% to 98% by geographic region, with adolescent digital IPV similarly variable in prevalence (5.8–92%).^4^, ^5^ Due to the absence of direct physical injury, digital IPV is often perceived by policymakers and the public as less harmful than in-person IPV.^6^ However, a growing body of research demonstrates that the unique affordances of digital platforms may exacerbate the psychological consequences of abuse, sometimes exceeding those of in-person IPV.^2^, ^7^, ^8^, ^9^

The emergence of digital IPV and rapid evolution of technology raise urgent questions about how digital contexts change the way adolescents engage in relationships and the long-term psychological and developmental consequences.^10^ Despite its growing prevalence and impact, digital IPV is overlooked in global IPV prevention and response strategies.^11^, ^12^ With less than five years until the current 2030 deadline of the United Nations (UN) Sustainable Development Goals (SDGs), now is an apt time to integrate digital IPV into global health priorities. This paper critically examines the global characteristics, risk factors, and consequences of digital IPV and urges its integration into international policy frameworks aimed at IPV prevention and intervention.

## How does the digital landscape change IPV?

The integration of digital technologies into adolescent romantic relationships has changed the dynamics of digital IPV. Tracking applications, spyware, text messages, social media, and intimate digital content now serve as tools through which harm can be perpetrated and experienced. Understanding how media affordances, i.e., the features of technologies that influence user behavior, interact with adolescent relationship dynamics is essential in addressing the evolving landscape of digital IPV.^13^ A key media affordance is the absence of interpersonal cues, such as facial expressions, body language, or vocal tone. This absence can distort the perception of hostility and reduce inhibitory control, making it easier for adolescents to perpetrate harm by simply typing a message or passively surveil without immediate social feedback or consequence; in other words, without adolescents having to see their partner hurt or upset.^9^, ^14^ Technology also affords remote control and monitoring, allowing perpetration without being physically present. Examples include questioning a partner’s shared location, regulating their online interactions, or surveilling their social media activity. The remote nature of digital IPV increases access between adolescents to create omnipresent conditions for harm.^2^ The digital environment also introduces publicity to interactions that would otherwise remain private. Harmful content such as threats, coercion, or intimate images can be captured and nonconsensually shared to an entire social network with a single click. The permanence of digital content further means that digital IPV can be relived indefinitely, compounding psychological harm and social humiliation. The affordance of anonymity further exacerbates risk in a way that makes it easier for perpetrators to manipulate their identities to trick or groom potential romantic partners without immediate detection.^15^ Together, these affordances create a digital context for IPV that is uniquely relentless, boundaryless, and difficult to escape, especially for adolescents whose social lives are mostly embedded in digital spaces.

## Who is experiencing and perpetrating digital IPV?

Across cultures, adolescent digital IPV has similar risk factors to in-person IPV, including well-documented factors like racial discrimination, heterosexism, poverty, housing instability, poor mental health, substance use, adverse childhood experiences, and exposure to violence.^8^, ^16^ These shared correlates indicate a level of continuity between digital and in-person IPV, yet media affordances create a distinct context that impacts who is at risk. Affordances like technology’s accessibility and user-friendliness makes it easier to perpetrate and become a victim, explaining why technology has been shown to introduce IPV into otherwise healthy adolescent relationships.^9^, ^17^ Technology has also normalized behaviors that can easily cross from care into control, such as location-sharing for safety becoming unhealthy monitoring or stalking. Interventions targeting adolescents stereotypically “at-risk” for in-person IPV may overlook those vulnerable for digital IPV perpetration.^9^, ^17^

Digital contexts also create unique social dynamics that impact risk of digital IPV. Notably, gender differences in prevalence of adolescent digital IPV are less pronounced than those of in-person IPV, which is reported by more girls and reflects well-documented patriarchal power structures embedded across global contexts. Conversely, the boundaries between victim and perpetrator are often blurred in digital contexts, with research cumulatively suggesting no gender difference in digital IPV victimization or that boys may be at greater risk, while girls experience more severe health impacts.^16^, ^18^ In addition, evidence shows that trans adolescents have a higher prevalence of digital IPV than cisgender adolescents in part because of their reliance on digital platforms for safe and authentic connections that may not be possible where they live.^19^ Similarly, technology may increase digital IPV risk for adolescents who are disabled or live in rural areas where technology provides critical access to romantic relationships.^18^ Technology also introduces adolescents to a global range of potential partners who can exploit the relative anonymity of technology to engage in harmful relationships with adolescents who might otherwise not engage with them.^15^

## What are consequences of digital IPV?

Adolescence reflects a sensitive period of transition, when teens’ brains drastically mature to help them explore new social experiences and acquire key social-emotional competencies. Experiencing both in-person and digital IPV during this developmental period are linked to myriad psychological consequences, including depression, anxiety, psychological distress, low self-esteem, self-harm, and suicide attempts.^2^ However, some psychological impacts are shown to be more strongly correlated with digital IPV compared to in-person IPV, including alcohol and substance misuse; difficulty sleeping; feelings of shame, helplessness, intrusion, and loneliness; and reduced quality of life. The heightened impact of digital IPV on mental health is thought to be the result of media affordances that alter the context within which teens are learning to manage communication and conflict in their relationships.^2^, ^7^, ^9^, ^14^ With limited interpersonal feedback via digital platforms, early evidence suggesting that adolescents who utilize digital communication with partners demonstrate reduced communication, adaptability, and conflict management skills.^9^, ^14^ The long-term impact of digital IPV on social-emotional skills has yet to be understood. Moreover, physical injury is not a direct consequence of digital IPV, but physical aggression may indirectly be caused by digital IPV due to the association between digital and in-person IPV.^20^ One longitudinal study found that digital IPV predicted physical aggression a year later. ^7^ Another cross-sectional study indicated that experiencing both in-person and digital IPV increased the risk of mental disorders compared to only in-person or only digital IPV, demonstrating that the impacts of digital and in-person IPV do not exist in silo.^21^

## What does this mean for prevention and intervention?

Traditional IPV prevention often fails to account for digital tactics, limiting the ability of adolescents and families to recognize digital IPV.^6^ As such, prevention programs have shown small effects in reducing digital perpetration and victimization, highlighting the limited capacity to address digital IPV relative to in-person IPV.^22^ Existing interventions often prioritize separation of partners, which leaves them ill-equipped for digital IPV, where physical proximity between partners is irrelevant.^17^ In fact, interventions that prioritize separation may inadvertently escalate harm because of the remote nature of technology. Breaking up and blocking an ex-partner may lead to retaliation; the use of online platforms provide ways to publicly shame, humiliate, or spread disinformation; or the creation of malicious fake accounts to continue harassment and/or cyberstalking.^23^ Similarly, in-person IPV perpetrators may turn to technology to instigate digital IPV after they are cut off from in-person access. Seemingly harmless acts, like sending continued and unexpected messages following separation, functions to assert control and an ongoing harmful presence in the ex-partner’s life.^17^ Accordingly, adolescents who experience digital IPV report a diminished sense of ever fully separating and feeling safe from their ex-partners, leaving service providers grappling with how to intervene.^2^, ^17^

## How can global frameworks play a role in digital IPV?

Despite its pervasive and serious nature, digital IPV remains largely absent from global policy frameworks. The relative novelty of technology in combination with the absence of direct physical injury leads policymakers, schools, families, and teens to underestimate the severity of harm.^6^ The widely used World Health Organization (WHO) definition of IPV does not include digital acts and the UN SDGs lack reference to digital IPV.^3^, ^24^ Initiatives such as UN Women and the Global Partnership for Action on Gender-Based Online Harassment and Abuse importantly address digital harm between adults or non-partnered peers, but apply gender and violence frameworks that overlook romantic contexts and adolescence as a developmental period.^16^, ^25^, ^26^ We recommend three critical steps in the development of global frameworks for digital IPV.

***Establish a specific taxonomy.*** Incorporating a specific taxonomy for digital IPV within existing global frameworks for IPV and online forms of harm is a crucial step toward legitimizing digital IPV and mobilizing research, advocacy, and policy. Existing and important frameworks focus on topics such as cyberbullying, online child sexual harassment and abuse, and in-person IPV. However, no frameworks explicitly address adolescent digital IPV. IPV initiatives, even those that make efforts to incorporate digital IPV, focus largely on adults, which ignores the developmental impact of IPV on adolescents.^27^ Further, technology-facilitated gender-based violence agendas forefront digital sexual violence as opposed to digital platforms used to perpetrate psychological dating violence.^27^ Similarly, online child sexual harassment and abuse initiatives focus on adults who use technology to perpetrate against minors, excluding non-sexual digital harm and peer to peer harm.^28^ Cyberbullying, which centers adolescent peer to peer behaviors, treats online harm between teens as peer aggression, existing outside of the romantic context (Fig. 1).^28^ The conflation of adolescent digital IPV within other categories of online and partner abuse obscures focus and contributes to its relative invisibility in both policy and practice.

**Fig. 1:**
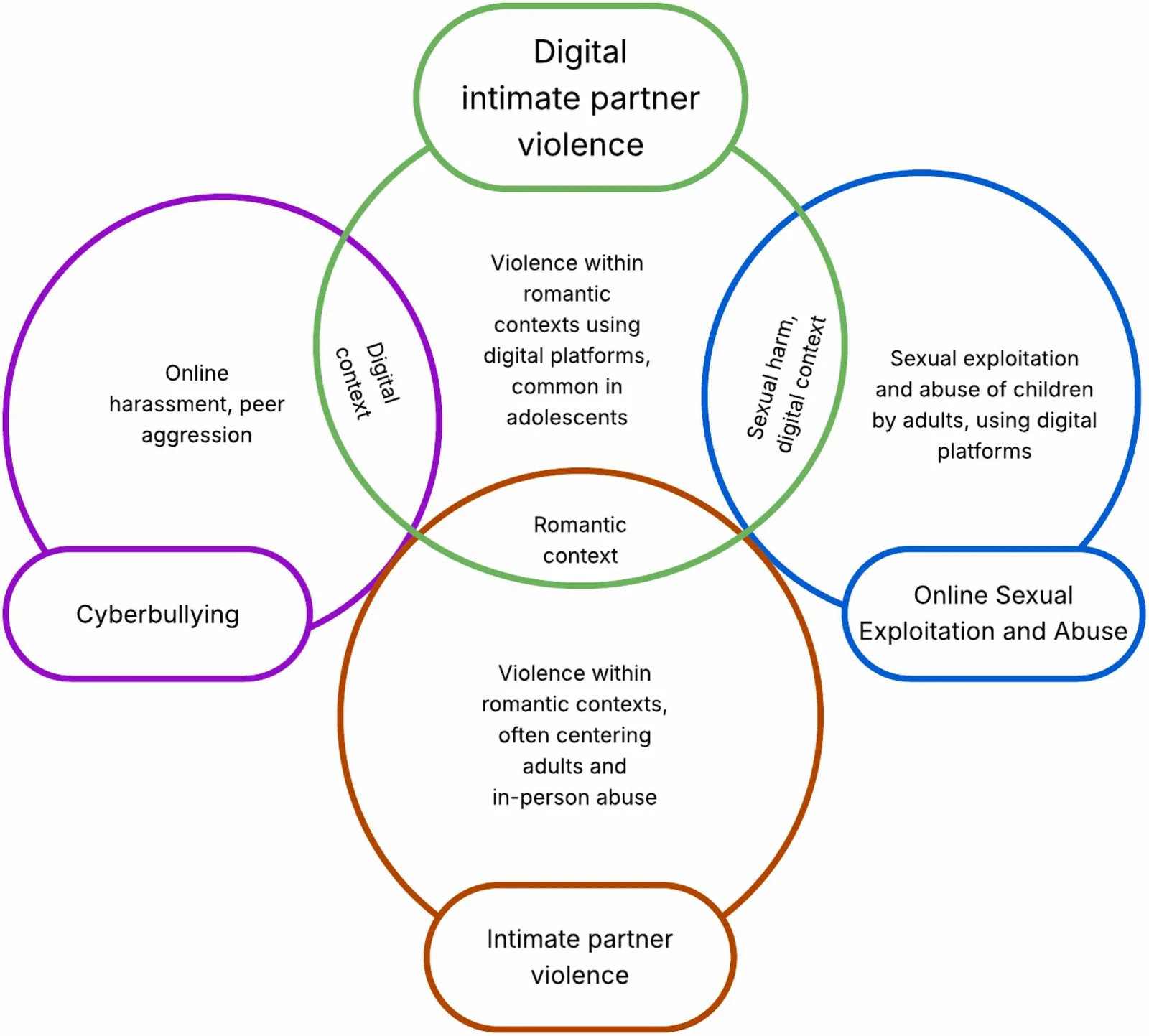
Overlapping frameworks with digital intimate partner violence.

Importantly, digital harm that occurs within the context of adolescent romantic relationships is often minimized and perceived as a normative part of adolescent romantic relationships. More overt forms of digital harm, such as types of cyberbullying or sexual harassment, do not look the same as the conversations between teen couples. Research suggests that digital IPV behaviors co-occur with affection, creating ambiguity that makes it even harder to recognize even though it causes emotional harm.^29^, ^30^ A taxonomy that intentionally considers adolescent digital IPV is vital to elevate the priority of addressing digital IPV among adolescents and recognizing the careful boundary between what is done out of care and what becomes harmful control.

Creating a taxonomy should involve establishing an international definition of digital IPV, a controlled and consistent vocabulary when describing digital IPV, and a clear system for organizing knowledge about digital IPV. Currently, the substantial variability in the language used to describe and define digital IPV across research, policy, and programming has created heterogeniety that hinders the ability to draw reliable and comparable of global prevalence estimates.^18^, ^31^, ^32^ By establishing international terminological and definitional clarity, global frameworks can promote international research collaboration, reduce inefficiencies across digital IPV research, and make it easier for researchers, practitioners, and families to access needed information about recognizing and preventing digital IPV. Moreover, clarifying a shared lexicon to use when defining and describing digital IPV is important to move forward in developing and validating scales capable of measuring the nuance and complexity of digital IPV across cultures so that we can all better understanding rates and patterns of digital IPV.

***Create global research agenda.*** Integrating adolescent digital IPV into global frameworks can also play a vital role in advancing research, including the development of a shared lexicon and agenda for researchers across the world. The rapid digitalization of adolescent relationships has outpaced scholarship in understanding the full scope, long-term impacts, and risk factors of digital IPV.^2^ Notably, existing screening tools typically intended to recognize in-person IPV are, by design, unable to detect digital IPV.^22^, ^32^ A systematic review of screening instruments found that, though tools specific to digital IPV are emerging, there remains a concerning lack of psychometric evidence of validity and reliability.^22^, ^32^ A global research agenda should prioritize developing measurement for digital IPV, including validating existing tools in global communities, increasing generalizability across cultures or adapting existing tools to other cultural contexts, and ensuring efficacy among adolescents. Research should also aim to both quantitatively and qualitatively explore and understand the lived experiences of adolescents who experience and perpetrate digital IPV across diverse cultural contexts. This will help clarify how culture influences digital IPV experiences. Longitudinal studies are additionally critical for assessing the long-term impacts of digital IPV as well as for clarifying the temporality of in-person and digital IPV among adolescents, which is particularly important because of trends that digital IPV may be a precursor to in-person IPV and that digital IPV may also introduce abusive behaviors into otherwise healthy relationships.^5^, ^9^, ^17^ Additional work is also needed to examine both risk and resilience factors behind perpetration of digital IPV compared to in-person IPV.

***Develop guiding principles for prevention and intervention.*** Global frameworks, led by organizations such as the WHO and UN, are critical for establishing international norms, informing legislation and programmatic responses, holding countries accountable, and facilitating global collaboration in strategic prevention and intervention efforts. Moreover, they can facilitate the translation of global policy into local actions, making it easier for providers, educators, families, and adolescents to access resources, guidance and information to prevent digital IPV.

## Recommended guiding principles

In order to appropriately address the sensitivity of adolescent digital IPV, we recommend the development of guiding principles in policy and practice that prioritize:

***Adolescents as a key risk group.*** Due to their frequent use of technology and the sensitive and transformative nature of adolescence as a developmental period, adolescents are high-risk for digital IPV. Adolescents are situated across the globe as early adopters of technology and consistently use digital platforms more than any other age groups.^33^, ^34^ Approximately four in five teens across the globe have internet access, consistently higher than any other age group regardless of region.^33^ Over a third of global adolescents report continuous digital contact with their friends throughout the day.^35^ In the US, it was estimated that 98% of older teens have smartphones.^36^ Not only do teens use technology frequently, but how they use technology to mediate romantic relationships is important in the development of their cognitive schemas.^1^ Romantic relationships experienced during adolescence influence the very neural processes and social-emotional competencies that impact relationship trajectories throughout their lives.^1^, ^37^ Adolescents who experience relational abuse develop schemas that normalize aggression as a conflict resolution tool, demonstrating the long-term consequences that digital IPV can have on adolescents.^37^
***The tension between autonomy and control during adolescence.*** Age restrictions are often a first response to preventing digital harm and reflect a valid desire to protect teens. Australia, for example, recently passed a historic ban of social media for adolescents under the age of 16 years.^38^ The argument can and has been made that age restrictions for social media are appropriate because, similar to R-rated movies, the restrictions are based on the highest risk content in a product that a young user may be harmed by rather than everything about the product is harmful.^39^ However, a key distinction from other types of media and social media is that social media is not merely a product that users consume, but also one that users use to communicate and interact with others. For example, in South Africa, social media has created a contemporary dating environment that enables adolescents girls to actively participate initiating relationships in cultural contexts where gendered power dynamics remain rigid.^40^ As such, restricting access does not only limit the potential for harm, it also limits opportunities for adolescents to build digital communication skills and navigate online relationship risks in an increasingly technology-mediated world. These are skills that will be used throughout adulthood that are formed during adolescence when the brain is uniquely prepared for these off-line and online socialization experiences.^41^ Teen participation in digital platforms, with the right tools and resources, can also help teach them digital competence, how to maintain healthy communication on social media, and learn to cope with the digital experience they will undoubtedly experience as adults.
***Co-creation with adolescents.*** Collaborating with adolescents can optimize the relevance and effectiveness of policies. Adolescents, as experts in their lived experience, have the fundamental right to participate in decision-making that affects them and the insight of how technology is being used by teens.^42^ Critically, the contextual factors of adolescents’ lives and communication patterns that influence recognition of digital IPV are needed to inform prevention and intervention.^30^ Adolescents bring an important contextual perspective to the table that is an integral part of identifying communication patterns that may otherwise go overlooked by adults. Partnering with adolescents is critical to forefront the development of effective strategies that recognize the digital tactics of IPV that teens are using in real life, such as surveillance, manipulation, threats, and humiliation as forms of IPV. Learning from and gaining feedback from adolescents about their experiences and perspectives can also aid in the co-creation of programming and policies that protect adolescents and are more likely to be appropriate, effective, and sustainable with the target audience. This includes the incorporation of how digital platforms are used by teens across cultural practices, to help distinguish harm from normal and healthy teen love across cultures.
***Partnership with technology.*** The rise of digital IPV exposes technology’s immense social influence. How teens establish and manage relationships is increasingly digitally mediated. Digital platforms are used by teens across the globe to maintain a level of constant connectivity with their partners and friends. However, the same platforms that adolescents use to communicate are often not built with their specific developmental needs in mind, resulting in teens using technology in ways that were not always the original intent of developers. Location sharing apps intended to keep friends and family safe can just as quickly turn into a tool for undue surveillance. The quick connection between partners enabled by instant messaging can also make being left on read feel like a punishment. Albeit not the intended use of these technologies, the ability to use digital platforms in harmful ways is a result of the design of the platform itself, and can thus be mitigated in the design stage. It is the responsibility of not just policymakers, practitioners, families, and researchers to step in and protect adolescents, but also that of the technology developers who are creating the digital platforms used to perpetrate harm and who also have the power to include safeguards in the design of digital platforms to mitigate the misuse of digital tools, such as incorporating age appropriate standards, strengthening age verification, removing addictive patterns from digital services, and using features that redirect adolescents towards in-person connection.^43^, ^44^ Truly, in order to effectively prevent adolescent digital IPV, a cohort of partners need to be in the room together, including technology developers who have the expertise and the power to make change at a design level and adopt protection standards that transcend borders to reduce and intervene in cases of digital IPV on a global scale in a way that state-level legislation cannot attain. Global frameworks created in conversation with technology developers is critical to establishing strategic guidance on the design and evaluation of technology to better protect adolescents against digital IPV. This includes the development of industry standards that integrate regulation into design practices to navigate moral and legal responsibility in the digital age. Building partnerships between global health organizations and technology corporations to develop age-appropriate standards can promote the development of technology that not only mitigates harm, but also creates safe digital environments capable of helping adolescents learn about and experience healthy love without violence in a way that balances the developmental tension between autonomy and protection.

## Conclusions

Adolescent digital IPV is an urgent and understudied global health concern with profound and lasting implications for health and development. The romantic and social experiences of teens create dynamics for adolescent digital IPV that fundamentally differ from those described in existing frameworks for cyberbullying, online sexual exploitation, or adult IPV. Incorporating digital IPV into existing international frameworks for public health and adolescent safety is key not only to validate its global significance, but to drive coordinated research, evidence-based programming, and adolescent-centered policies to combat digital IPV and develop technology to foster resilience and healthy relationship development worldwide. Where technology has transformed the way adolescents experience harm in their relationships, it also offers opportunities to deliver innovative solutions. Digital tools can be designed to expand access to education and support, help teens and families recognize and address digital IPV, and equip school staff and health providers with resources and guidance needed to screen for and respond to digital IPV. By elevating technology as more than solely a source of risk but as a vector for creating safe havens for adolescents to build safe, supportive, and developmentally appropriate digital environments, we can not only protect teens from harm, but also help them understand, experience, and learn what healthy love feels like in a digitally mediated world.

## CRediT authorship contribution statement

**Taren L. McGray:** Writing - review & editing, Writing - original draft, Conceptualization; **Thao Ha:** Writing - review & editing, Funding acquisition, Conceptualization.

## Funding Disclosure

This publication was supported by the 10.13039/100000025National Institute of Mental Health of the 10.13039/100000002National Institutes of Health under Award Number RF1MH138928 awarded to Dr. Ha and supporting Dr. McGray. The content is solely the responsibility of the authors and does not necessarily represent the official views of the National Institutes of Health and the National Institute of Mental Health.

## Declaration of Generative AI and AI-assisted technologies in the writing process

Generative AI was not used in the preparation of this manuscript.

## Declaration of Competing Interests

The authors declare that they have no known competing financial interests or personal relationships that could have appeared to influence the work reported in this paper.

## Data Availability

No data was used for the research described in the article.
